# Adipsin inhibits Irak2 mitochondrial translocation and improves fatty acid β-oxidation to alleviate diabetic cardiomyopathy

**DOI:** 10.1186/s40779-023-00493-5

**Published:** 2023-12-11

**Authors:** Meng-Yuan Jiang, Wan-Rong Man, Xue-Bin Zhang, Xiao-Hua Zhang, Yu Duan, Jie Lin, Yan Zhang, Yang Cao, De-Xi Wu, Xiao-Fei Shu, Lei Xin, Hao Wang, Xiao Zhang, Cong-Ye Li, Xiao-Ming Gu, Xuan Zhang, Dong-Dong Sun

**Affiliations:** 1 Department of Cardiology, Xijing Hospital, Air Force Medical University, Xi’an, 710032 China; 2 Department of Basic Medicine, Air Force Medical University, Xi’an, 710032 China; 3 Department of Physiology and Pathophysiology, Air Force Medical University, Xi’an, 710032 China; 4 Institute for Hospital Management Research, Chinese PLA General Hospital, Beijing, 100853 China

**Keywords:** Diabetic cardiomyopathy, Mitochondrial translocation, Mitochondrial function, Fatty acid β-oxidation

## Abstract

**Background:**

Diabetic cardiomyopathy (DCM) causes the myocardium to rely on fatty acid β-oxidation for energy. The accumulation of intracellular lipids and fatty acids in the myocardium usually results in lipotoxicity, which impairs myocardial function. Adipsin may play an important protective role in the pathogenesis of DCM. The aim of this study is to investigate the regulatory effect of Adipsin on DCM lipotoxicity and its molecular mechanism.

**Methods:**

A high-fat diet (HFD)-induced type 2 diabetes mellitus model was constructed in mice with adipose tissue-specific overexpression of Adipsin (Adipsin-Tg). Liquid chromatography-tandem mass spectrometry (LC–MS/MS), glutathione-S-transferase (GST) pull-down technique, Co-immunoprecipitation (Co-IP) and immunofluorescence colocalization analyses were used to investigate the molecules which can directly interact with Adipsin. The immunocolloidal gold method was also used to detect the interaction between Adipsin and its downstream modulator.

**Results:**

The expression of Adipsin was significantly downregulated in the HFD-induced DCM model (*P* < 0.05). Adipose tissue-specific overexpression of Adipsin significantly improved cardiac function and alleviated cardiac remodeling in DCM (*P* < 0.05). Adipsin overexpression also alleviated mitochondrial oxidative phosphorylation function in diabetic stress (*P* < 0.05). LC–MS/MS analysis, GST pull-down technique and Co-IP studies revealed that interleukin-1 receptor-associated kinase-like 2 (Irak2) was a downstream regulator of Adipsin. Immunofluorescence analysis also revealed that Adipsin was co-localized with Irak2 in cardiomyocytes. Immunocolloidal gold electron microscopy and Western blotting analysis indicated that Adipsin inhibited the mitochondrial translocation of Irak2 in DCM, thus dampening the interaction between Irak2 and prohibitin (Phb)-optic atrophy protein 1 (Opa1) on mitochondria and improving the structural integrity and function of mitochondria (*P* < 0.05). Interestingly, in the presence of *Irak2* knockdown, Adipsin overexpression did not further alleviate myocardial mitochondrial destruction and cardiac dysfunction, suggesting a downstream role of Irak2 in Adipsin-induced responses (*P* < 0.05). Consistent with these findings, overexpression of Adipsin after *Irak2* knockdown did not further reduce the accumulation of lipids and their metabolites in the cardiac myocardium, nor did it enhance the oxidation capacity of cardiomyocytes expose to palmitate (PA) (*P* < 0.05). These results indicated that Irak2 may be a downstream regulator of Adipsin.

**Conclusions:**

Adipsin improves fatty acid β-oxidation and alleviates mitochondrial injury in DCM. The mechanism is related to Irak2 interaction and inhibition of Irak2 mitochondrial translocation.

**Supplementary Information:**

The online version contains supplementary material available at 10.1186/s40779-023-00493-5.

## Background

The prevalence of diabetes mellitus is increasing rapidly worldwide and has become one of the serious public health problems [1, 2]. Cardiac dysfunction, a common sequela of diabetes, is considered a major cause of morbidity and mortality in patients with diabetes mellitus [3]. Among them, progressive structural and functional remodeling of the heart unrelated to coronary arteries and hypertension, is termed diabetic cardiomyopathy (DCM) [4]. Similar to the clinical classification of diabetes mellitus, the DCM models used in the study are also divided into type 1 and type 2, with type 2 accounts for more than 90% of human diabetic patients [5]. However, effective therapeutic interventions for type 2 DCM are currently limited.

DCM, as a metabolic disease, initially exposes the heart to a high-fat lipotoxic environment, which leads to its pathophysiology [6]. The diabetic heart maintains cardiac output by increasing the utilization of fatty acids, which is thought to be an adaptive process [7, 8]. In the pathogenesis of DCM, the excessive utilization and uptake of fatty acids and lipids exceed the adaptive regulation, leading to the accumulation of fatty acids and lipids in cardiomyocytes, eventually producing lipotoxicity and affecting myocardial contractile function [9].

Adipocytes are considered active endocrine organs and secrete a variety of bioactive factors called adipokines, which are mainly used to control the metabolism of fat homeostasis [10]. Dysregulation of adipokines is an important mechanism of metabolic diseases [11]. A large body of evidence suggests a strong link between adipokines and increased cardiovascular risks in obese patients [12, 13]. In recent years, adipokine-mediated crosstalk between adipose tissue and the heart has been widely recognized [14]. Normal physiological levels of multiple adipokines are essential to maintenance of normal cardiovascular function [15, 16]. Among the numerous adipokines, Adipsin has attracted attention due to its reduced levels in animal models of obesity and type 2 diabetes mellitus [17]. Our previous work showed that Adipsin has a cardioprotective role in myocardial viability, cardiac dysfunction and adverse remodeling during myocardial infarction (MI) injury [18]. Adipsin is mainly encapsulated in extracellular vesicles and taken up by target cells, thereby exerting its respective effects [19]. The aim of this study is to investigate the effect of Adipsin on DCM induced by high-fat diet (HFD) and its possible mechanisms.

In particular, we constructed an animal model with adipose tissue overexpression of Adipsin to evaluate whether Adipsin has cardioprotective effects. This study also aimed to examine how Adipsin regulates myocardial metabolism and thus affects myocardial function and its specific molecular mechanisms. Our observation helped elucidate a novel mechanism of Adipsin alteration in DCM pathophysiology, revealing the reversal of mitochondrial dysfunction as a therapeutic target for DCM.

## Materials and methods

### Animal

All animal experiments were carried out in accordance with the guidelines for the use of experimental animals of the National Institutes of Health and were approved by the Animal Experiment Ethics Committee of Air Force Medical University (No. 20201017). The Cre-LoxP system was used to regulate the expression of the target gene *Adipsin.* A recombinant vector Rosa26-(SA/PCAG)-loxp-stop-loxp-cDNA-PA was constructed and inserted into the first intron of Rosa26. The knock-in mice were crossed with adipose-specific Cre mice (Adipoq-cre) to generate a conditional overexpression mouse model with tissue-specific expression of foreign genes. The obtained Rosa26LSL/+ :Cre was successfully constructed as adipose tissue-specific overexpression mice, and the transgenic mice were named Adipsin-Tg, while the Rosa26LSL/+ mice failed to construct adipose tissue-specific overexpression Adipsin and were named nontransgenic mice (NTg) [20]. Eighty 6-week-old male C57BL/6J mice were obtained from the Animal Center of the Air Force Medical University (Xi’an, China), and divided into 4 groups: Adipsin-Tg mice (*n* = 32), NTg mice (*n* = 32), and wild-type (WT) mice (C57BL/6, *n* = 16). In addition, newborn mice (1–3 days old, *n* = 20), and adult mice (8–12 weeks old, *n* = 10) were also used in the present study.

These mice were given a normal chow diet (CHD) or a high-fat diet (HFD), and were divided into CHD + Adipsin-Tg group (*n* = 16), CHD + NTg group (*n* = 16), CHD + WT (*n* = 16), HFD + Adipsin-Tg (*n* = 16), and HFD + NTg (*n* = 16) group. Diabetic mice were fed with a HFD (5.24 kcal/g; 60% of calories from fat, 20% of calories from protein, and 20% of calories from carbohydrate; D12492, Research Diets, USA), whereas control mice received a CHD (3.85 kcal/g; 10% of calories from fat, 20% of calories from protein, and 70% of calories from carbohydrate; D12450J, Research Diets, USA). Mice were kept in a light/dark cycle at 23 °C for 12 h and given adequate water. Six-week-old Adipsin-Tg or NTg mice were given a HFD for 6 months to establish a DCM mouse model for subsequent experiments [21]. Target gene expression was downregulated in mouse myocardium by myocardial spot injection. Anesthesia with 2% N-isoflurane was used during the operation process. After exposing the heart, adenovirus (1 × 10^10^ GC/ml, 40 μl per mouse) (50 μl needle, 705RN, Hamilton, USA) was injected into the free wall of the left ventricle. White adipose tissue (WAT), brown adipose tissue (BAT), heart, lungs, spleen, liver, kidney, and muscle tissues were then rapidly removed for subsequent experiments. At the end of the experiment, mice were euthanized using 30% VD/min of 100% carbon dioxide in accordance with the 2020 AVMA Euthanasia Guidelines.

### Primary isolation and culture of neonatal and adult mouse cardiomyocytes, neonatal fibroblasts and endothelial cells

As described earlier [22], the chest wall of newborn mice (1–3 days old, *n* = 20) was dissected and the hearts were removed. The ventricles were cut into pieces and digested with 0.1% type II collagenase. After centrifugation, the precipitate containing cardiomyocytes and cardiac fibroblasts was preserved, and primary cardiomyocytes and cardiac fibroblasts were obtained by differential adherence method. For primary endothelial cells, we sequentially digested with 0.2% collagenase II and 0.25% trypsin in the ventricle, followed by centrifugation at 1000 *g* for 5 min to produce endothelial cell precipitates [19]. Adult mice (8–12 weeks old, *n* = 10) were anesthetized with N-isoflurane (2%) and hearts were rapidly excised. The hearts were perfused using the Langendorff system (PanLab, Barcelona, Spain), and cardiomyocytes were isolated and cultured according to previously described methods [23].

### Immunogold staining and transmission electron microscope analysis

For immunocolloidal gold electron microscopy studies, mice were anesthetized with N-isoflurane (2%) and hearts were rapidly excised. Heart tissue samples with a volume of less than 1 mm^3^ were rapidly fixed in an immunocolloidal gold electron microscopy fixative (G1124-100ML, Servicebio, China). Samples were then dehydrated with gradient ethanol, wrapped in resin, polymerized and sectioned with an ultraminiform microtome (70–80 nm), and coated with nickel mesh foil. The grids were hydrated with phosphate buffer saline (PBS) and blocked with PBS containing 0.1% bovine serum albumin (BSA) to block nonspecific reactions. Anti-interleukin (IL)-1 receptor-associated kinase-like 2 (Irak2) antibodies (bs-1427R, Bioss, China) were diluted to 1:500 with blocking solution and incubated overnight at 4 °C. Samples were then rinsed with trisbuffered saline (TBS) at room temperature followed by incubation with secondary antibody (10 nm colloidal gold goat anti-mouse, G7777, Sigma-Aldrich, Germany). Samples were fixed with 1% glutaraldehyde in PBS for 7 min and incubated with acetate uranyl and lead citrate. Images were acquired under a transmission electron microscope (HT7700, HITACHI, Japan). Black gold particles (10 nm in size) were considered as positive expression [24].

For routine transmission electron microscope examination, fresh heart tissue with a volume less than 1 mm^3^ was quickly placed into an electron microscope fixation device (G1102, Servicebio, China) for internal fixation, rinsed, re-fixed, dehydrated in a gradient, permeable, embedded, and cut into 60–80 nm ultrathin sections with an ultrathin microtome. Images were obtained with a transmission electron microscope (HT7700, HITACHI, Japan) at 300 kV. Mitochondrial images were analyzed using ImageJ software [25].

### Measurement of mitochondrial oxygen consumption rate (OCR)

A Seahorse XF Cell Mitochondrial Stress Test kit (103015-100, Agilent, USA) was used. In brief, primary mouse cardiomyocytes were seeded at a density of 1 × 10^5^/ml in Agilent Seahorse XF24 cell culture microplate (V7-PS, 100777-004, Agilent, USA) on an XF24 extracellular flux analyzer (Agilent Seahorse Bioscience, USA). The Seahorse XF Cell Mitochondrial Stress Test kit was used to provide inhitiors in sequence on plates containing culture medium, including oligomycin (1 μmol/L), fluoro-carbonyl cyanide phenylhydrazone (FCCP; 0.5 μmol/L), rotenone (1 μmol/L), and antimycin A (1 μmol/L). Wave software (Agilent Seahorse Bioscience, USA) was employed for data collection and calculated basic and maximum OCR and other parameters [26].

### Small interfering RNA (siRNAs) and recombinant adenovirus vectors construction

#### Construction of *Irak2* siRNAs and recombinant adenovirus vector expressing Irak2 short hairpin RNA (shRNA)

To downregulate *Irak2* expression, three *Irak2* siRNAs were designed by BLOCK-iT™ RNAi Designer and synthesized by Tsingke Biotechnology Company (Beijing, China) (Additional file 1: Table S1). For in vitro studies, primary cardiomyocytes were transfected with Lipofectamine 2000 (Thermo Fisher Scientific, Michigan, USA), the one with the highest knockdown efficiency was selected to convert the selected siRNA sequence to an shRNA sequence. The empty adenovirus vector (Ad-shControl) and the recombinant adenovirus vector expressing shIrak2 (Ad-shIrak2) were constructed by Tsingke Biotechnology Company (Beijing, China). The sequences of shRNA for *Irak2* were 5′-CCGGGCACCTTTGCCGATATCTCTCGAGTTTTTT-3′ and 5′-AATTAAAAAAGCACCTTTGCCGATATCTCTCGAGAGATATCGGCAAAGGTGC-3′. In vivo experiments, the target gene *Irak2* was introduced into the hearts of Adipsin-Tg or NTg mice by myocardial spot injection (NTg + Ad-shControl, NTg + Ad-shIrak2, Adipsin-Tg + Ad-shControl, Adipsin-Tg + Ad-shIrak2).

#### Construction of cell line overexpressing Adipsin

To enhance *Adipsin* expression, Adipsin was delivered in vitro using adenovirus carrying Adipsin (Ad-Adipsin) and a control vector (Ad-Control). After cells had grown to 50–60% confluence as described previously [27], adenovirus was transduced into cardiomyocytes at 100:1 multiplicity of infection in FBS-free medium, and various subsequent experiments were performed.

### Echocardiography

M-mode echocardiography was performed using a VEVO 2100 echocardiography system (VisualSonics Inc., Toronto, Canada). Briefly, mice were anesthetized with 2.5% isoflurane. Anesthesia was maintained by inhalation of 2% isoflurane, and heart rate was recorded (approximately 400 bpm). End-systolic left ventricular internal diameters (LVIDs) and end-diastolic left ventricular internal diameters (LVIDd) were measured. Left ventricular ejection fraction (LVEF) and left ventricular fractional shortening (LVFS) were calculated by software algorithm. Early diastolic (E) and late diastolic (A) mitral cross flow velocity and the ratio of early diastolic (E) to late diastolic (A) mitral flow velocity (E/A) were measured by Doppler echocardiography. Pulsed-wave Doppler images of mitral E-wave and A-wave velocities was obtained from a four-chamber cardiac view. The E/A ratio was measured to evaluate diastolic function. The parameters were derived from five consecutive cardiac cycles in M-mode. Early-diastolic peak velocity (e’) of mitral valve ring was also measured, and E/e’ was calculated to reflect left ventricular diastolic function. All echocardiographic images were analyzed with the use of the Vevo 2100 software (Toronto, Canada). Vevo Strain Software (Toronto, Canada) was used to perform speckle tracking-based strain analyses of the parasternal long-axis B-mode loops [22].

### Real-time quantitative PCR

Total RNA was extracted from WAT, BAT, heart, lung, spleen, liver, kidney and muscle tissue, as well as cardiomyocytes, fibroblasts and endothelial cells using TRIzol (Invitrogen, 15596026, USA) and methylene chloride. Quantification was performed with Nano Drop2000 nucleic acid (Thermo Fisher Scientific, Michigan, USA). cDNA was then synthesized with the PrimeScript RT reagent kit and gDNA Eraser (rr047q, TaKaRa, Japan), and real-time quantitative-PCR was carried out by SYBR Premix Ex Taq II (rr820l, TaKaRa, Japan). All procedures were performed in accordance with the manufacturer’s guidelines [27]. Primer sequences are shown in the Additional file 1: Table S2.

### Co-immunoprecipitation (Co-IP)

Cardiomyocytes antigen samples were prepared using protein A/G immunoprecipitation magnetic beads (B23201, Bimake, USA), preprocessed with magnetic beads, conjugated with anti-Adipsin and anti-Irak2 antibodies, precipitated and eluted antigens. Experiments were performed according to the manufacturer’s protocol. Finally, 20–50 μl of 1 × sodium dodecyl sulfate–polyacrylamide gel electrophoresis (SDS–PAGE) loading buffer was added to the prepared antigen samples, thoroughly stirred, heated at 95 °C for 5 min. Magnetic separation was performed again. The supernatant was collected and placed in a new Eppendorf tube. After centrifugation at 13,000 *g* for 10 min at room temperature, the supernatant was collected for subsequent detection [18].

### Western blotting

Proteins or mitochondria were extracted from heart, epididymal WAT, BAT, etc., then protein loading buffer was added, boiled and subjected to electrophoresis by 10% or 12% SDS–PAGE (Cwbiotech, Beijing, China). Samples were transferred to 0.22-μm polyvinylidene difluoride absorbent membranes (Millipore, MA, USA). Membranes were blocked with 10% milk for 1 h at 37 °C and then incubated with specified primary antibodies overnight at 4 °C, followed by incubation with the corresponding secondary antibody at room temperature. To visualize the enhanced chemiluminescence (ECL) color bands using chemiluminescence technology. Densitometry was performed using ImageJ software. Protein density was normalized by β-actin, voltage-dependent anion channel 1 (VDAC1) and transferrin (TF) levels [27]. The primary antibodies used for Western blotting are shown in the Additional file 1: Table S3.

### Glutathione-S-transferase (GST) pull down and mass spectrometry analysis

Experiments were performed using the pierce GST Protein Interaction Pull Down kit (21516, Thermo Fisher Scientific, USA) according to the manufacturer’s manual. Firstly, the GST-tagged fusion protein GST-Adipsin (pET-21b + BamHI + Adipsin + GST + HindIII) was constructed as a protein bait (constructed and synthesized by Tsingke Biotechnology Company, Beijing, China). Prey proteins for primary cardiomyocyte lysis were obtained by immobilizing and binding bait proteins to immobilized bait proteins using balanced glutathione agarose. Then, the eluted protein complexes were employed for mass spectrometry analysis and subsequent immunoblotting [28].

### Immunohistochemistry

Mouse hearts were fixed overnight in 4% paraformaldehyde (pH 7.4), embedded in paraffin, and serially sectioned (5-mm-thick sections) for histological analysis. Sections were deparaffinized and endogenous peroxidase was blocked with antigen repair. After sealing with 3% BSA for 1 h, sections were incubated overnight with the specified primary antibodies in a wet box at 4 °C, followed by incubation with the corresponding secondary antibodies (HRP-labeled) and staining using diaminobenzidine (DAB) and hematoxylin. After dehydration and sealing, sections were visualized and images were collected. DAB-positive expression was brown yellow. At least 10 fields were randomly selected from each heart for immunohistochemistry analysis [29].

### JC-1 staining and flow cytometry

To detect mitochondrial membrane potential, cardiomyocytes were incubated with JC-1 (Beyotime, Shanghai, China) for 20 min at 37 °C. Cardiomyocytes were analyzed within 1 h using flow cytometry (CytoFLEX LX, Beckman, USA). All data were analyzed using EXPO32 ADC analysis software (Beckman, Washington, USA). For flow cytometry analysis, unstained cells were used as controls to identify the forward scatter/side scatter (FSC/SSC) gates used in the same experiments. A total of 10,000 cells was detected in each sample [30].

### Determination of malondialdehyde (MDA) and triglyceride (TG) content

The lipid oxidation MDA assay kit was used to detect the level of MDA in cardiac tissues (BC0025, Solarbio, China) [31]. Tissue TG assay kit (BC0625, Solarbio, China) was employed to monitor TG content in cardiac myocardium [32]. MDA and TG contents were calculated according to standard curve.

### Fatty acid β-oxidation (FAO) analysis

In adult mouse cardiomyocytes, FAO was measured according to the previously described method [21]. In brief, isolated adult cardiomyocytes were incubated with analysis medium containing fatty acids for 10 min, and at the end of the reaction the supernatant was added to 4 ml scintillation cocktail (Sigma-Aldrich, Germany) and radioactivity was measured using a scintillation counter [33].

### XF palmitate (PA) oxidative stress test

FAO levels were determined using the Seahorse XF PA Oxidative Stress Test kit (102720-100, Agilent, USA). Mouse adult cardiomyocytes were incubated at a density of 1 × 10^5^/ml in Agilent Seahorse XF24 Cell Culture Microplates (V7-PS, 100777-004, Agilent, USA). OCR was measured in the presence and absence of Etomoxir (HY-50202, MCE, USA), an inhibitor of carnitine palmitoyltransferase-1 (CPT-1), which is essential for FAO and is located at the inner surface of the outer mitochondrial membrane. Cells were stored overnight at 37 °C according to the manufacturer’s FAO assay protocol (Agilent, CA, USA). The PA oxidative stress test was performed in the presence of Etomoxir and PA-BSA or control-BSA, and each substance was added 0 and 15 min before the start of the assay, respectively. The final concentrations of the drugs used were: etomoxir 4 µmol/L, oligomycin 1.5 µmol/L, FCCP 1 µmol/L, and rotenone/antimycin A 0.5 µmol/L, respectively. FAO was determined by calculating the difference in OCR between Etomoxir treated and untreated cells. OCR values were expressed as pmol/(min·µg) protein [34].

### Enzyme-linked immunosorbent assay (ELISA)

Serum Adipsin levels were detected using the mouse Adipsin ELISA kit according to the manufacturer’s procedure (E-ELM0335c, Elabscience, China). The absorbance (OD) value was measured at 450 nm by spectrophotometer.

### Mitochondria biological function

To assess the functional status of mitochondria, citrate synthase (CS) and electron transport chain complex activities (complexes I/II/III/IV/V) were measured using commercially available kits (Sigma-Aldrich, Germany).

### Statistical analysis

Data are expressed as mean ± standard deviation (SD). SPSS software (version 23; Inc., Chicago, IL, USA) and GraphPad Prism 8 (GraphPad Software, La Jolla, CA, USA) software were used. Statistical analyses between groups were done by two-tailed Student’s *t* test or one-way ANOVA, with Fisher’s post hoc comparison test when appropriate. *P* < 0.05 was considered to be statistically significant.

## Results

### Adipsin is significantly downregulated in HFD-induced DCM

**Fig. 1:**
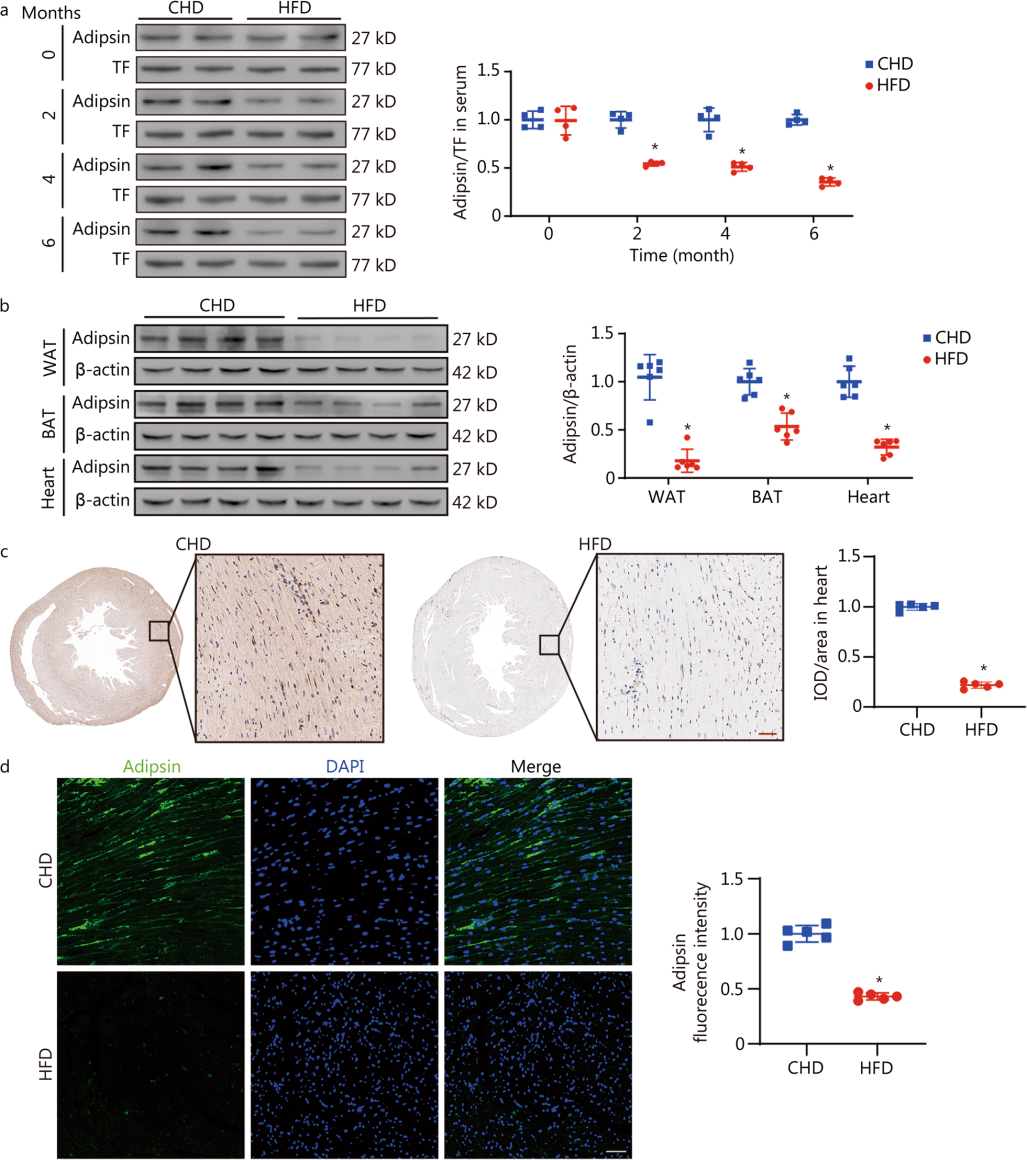
Adipsin is significantly downregulated in HFD-induced DCM. **a** Western blotting results and quantitative analysis of serum Adipsin levels in the treatment groups at 0, 2, 4, and 6 months (*n* = 4). **b** Western blotting results and quantitative analysis of Adipsin expression in WAT, interscapular BAT and heart tissue lysates from CHD- and HFD-fed mice (*n* = 6). **c**, **d** Immunohistochemical/immunofluorescence stainings and quantitative analysis of Adispin in mouse hearts from CHD- and HFD-fed groups (*n* = 5). Scale bar = 50 μm. Statistical significance was determined by two-tailed Student’s *t* test. All data are represented with mean ± SD. ^*^P < 0.05 vs. CHD; CHD chow diet, HFD high-fat diet, WAT white adipose tissue, BAT brown adipose tissue, TF transferrin, IOD integrated option density, DAPI 4’,6-diamidino-2-phenylindole

To identify the possible role of Adipsin in the pathophysiology of DCM, we used a mouse model of HFD-induced DCM (Additional file 1: Tables S4, S5). The serum Adipsin level began to decrease at the 2nd month of HFD feeding and reached the lowest level at the 6th month (*P* < 0.05; Fig. 1a, Additional file 1: Fig. S1a). After 6 months of HFD feeding, the expression of *Adipsin* mRNA was decreased in both WAT and BAT compared with CHD mice (WAT: 6.29 ± 0.45 vs. 10.79 ± 0.39, *P* < 0.05; BAT: 4.34 ± 0.23 vs. 7.63 ± 0.28, *P* < 0.05; Additional file 1: Fig. S1b). However, at 6 months, *Adipsin* mRNA expression was largely undetectable in heart, lung, spleen, liver, kidney and muscle tissues, as well as cardiomyocytes, cardiac fibroblasts and endothelial cells of CHD and HFD-fed mice (Additional file 1: Fig. S1b, c). In addition, Western blotting showed that Adipsin levels in WAT, BAT, and heart of DCM mice were significantly lower than those of normal mice (WAT: 0.18 ± 0.12 vs. 1.00 ± 0.24, *P* < 0.05; BAT: 0.54 ± 0.14 vs. 1.00 ± 0.14, *P* < 0.05; heart: 0.32 ± 0.08 vs. 1.00 ± 0.16, *P* < 0.05; Fig. 1b). Therefore, we speculated that adipose tissues express and secrete the adipokine Adipsin, which enters the diabetic heart and plays an important protective role. In support of these findings, immunohistochemical staining and immunofluorescence staining of myocardial tissues showed that the content of Adipsin was significantly decreased in HFD-fed mice (*P* < 0.05; Fig. 1c, d). These results suggest that Adipsin play an important role in the pathophysiology of DCM.

### Overexpression of Adipsin can improve cardiac function, reduce mitochondrial cristae damage, and improve mitochondrial function

To further examine the effects of Adipsin on DCM, heterozygous mice with conditionally overexpressing Adipsin were generated. Primers 1 and 2 were used to amplify the mouse genome. In WT mice, primer 1 showed a positive band at 994 bp and primer 2 showed a negative band at 749 bp. Heterozygous mice (Rosa26LSL/+) had a positive band at both primer 1 (994 bp) and primer 2 (749 bp). Heterozygous mice (Rosa26LSL/+) were crossed with adipose tissue-specific Cre mice (Adipoq-cre) to generate Adipsin-Tg (Rosa26LSL/+ :Cre) and their corresponding control mice (NTg). Adipsin-Tg (Rosa26LSL/+ :Cre) mice showed a positive band at primer Cre (400 bp) (Additional file 1: Fig. S1d). Compared with NTg mice, Adipsin mRNA levels in WAT and Adipsin protein levels in WAT and cardiac myocardium were significantly increased in Adipsin-Tg mice (*P* < 0.05; Additional file 1: Fig. S1e, f).

**Fig. 2:**
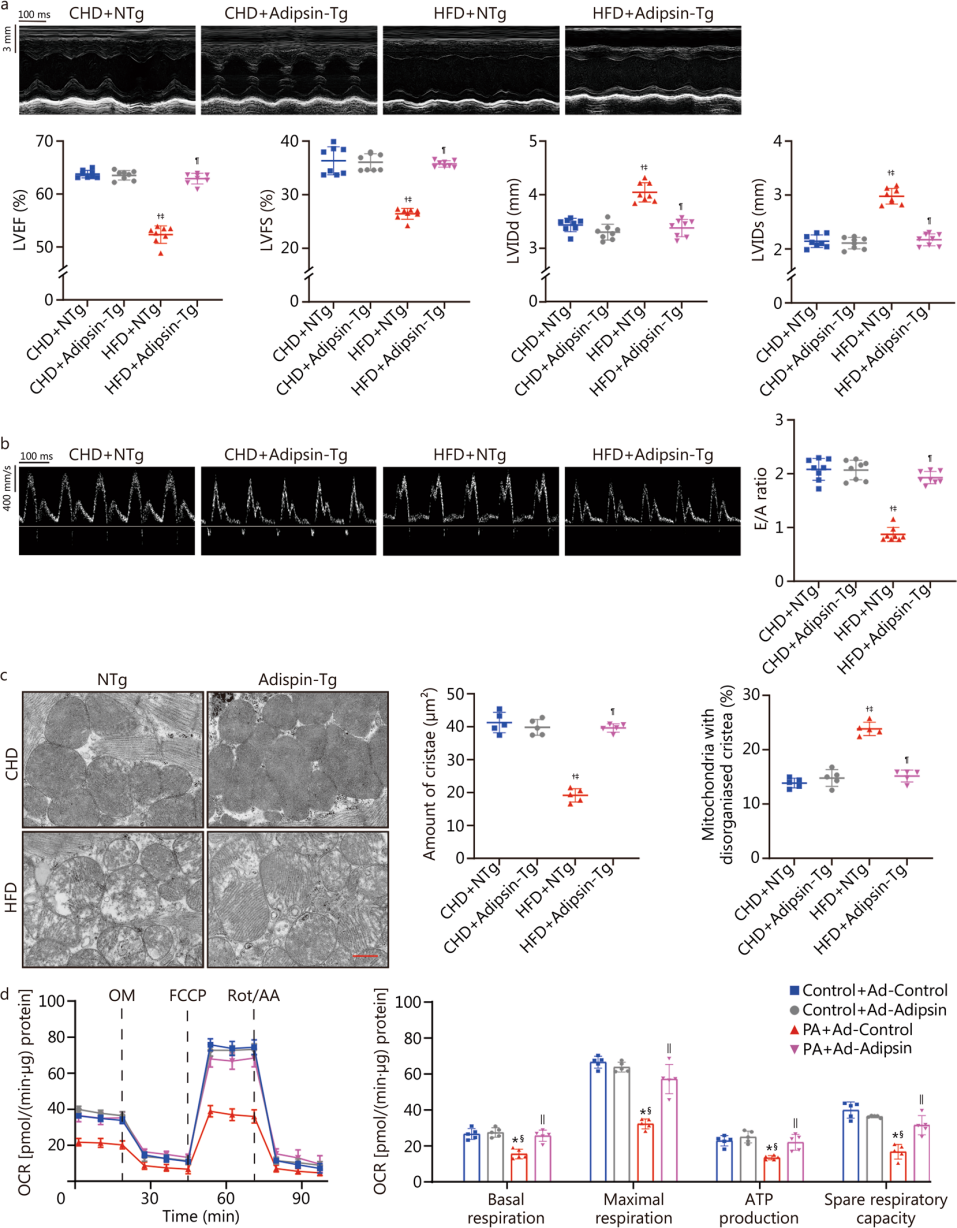
Overexpression of Adipsin can improve cardiac function, reduce mitochondrial cristae damage, and improve mitochondrial function. **a** Echocardiographic results and quantitative analysis of echocardiographic data including LVEF, LVFS, LVIDd and LVIDs (*n* = 8). **b** Pulse-wave Doppler results and quantitative analysis of the E/A ratio (*n* = 8). **c** Transmission electron microscopic images, and quantitative analysis of cristae amount per μm^2^ and the proportion of mitochondria with disorganized cristae of myocardium from NTg and Adipsin-Tg groups with CHD- or HFD-feeding (*n* = 5). Scale bar = 500 nm. **d** OCR in cardiomyocytes from various treatment groups, and quantitative statistical analysis of OCR including basal respiration, maximal respiration, ATP production, and spare respiration capacity. Statistical significance was determined using one-way ANOVA analysis. All data are represented with mean ± SD. ^†^*P* < 0.05 vs. CHD + NTg; ^‡^*P* < 0.05 vs. CHD + Adipsin-Tg; ^¶^*P* < 0.05 vs. HFD + NTg; ^*^P < 0.05 vs. Control + Ad-Control; ^§^*P* < 0.05 vs. Control + Ad-Adipsin; ^||^*P* < 0.05 vs. PA + Ad-Control. CHD chow diet, HFD high-fat diet, Adipsin-Tg Adipsin tissue-specific transgenic mice, NTg nontransgenic mice, LVEF left ventricular ejection fraction, LVFS left ventricular fractional shortening, LVIDd end-diastolic left ventricular internal diameters, LVIDs end-systolic left ventricular internal diameters, E/A the ratio of mitral peak velocity of early filling (E) to mitral peak velocity of late filling (A), OCR oxygen consumption rate, OM oligomycin, FCCP fluoro-carbonyl cyanide phenylhydrazone, Rot/AA rotenone and antimycin A, PA palmitate

Echocardiographic analysis showed that LVEF, LVFS, and E/A ratio of HFD-fed mice were significantly decreased, and LVIDd and LVIDs were increased (*P* < 0.05; Fig. 2a, b), indicating that both systolic and diastolic functions were severely impaired in DCM mice. Adipsin overexpression reversed these effects. These results indicate that Adipsin improves cardiac function in DCM mice. In addition, swollen and structurally abnormal myocardial mitochondria were observed in DCM mice compared with control mice. Adipsin-Tg diabetic mice showed reduced cardiomyocyte mitochondrial structure damage, evidenced by mitochondrial cristae fragmentation (*P* < 0.05; Fig. 2c). In this context, Adipsin attenuates myocardial mitochondrial cristae damage and preserves mitochondrial structure. Moreover, Adipsin significantly improved mitochondrial complex I/II/III/IV/V activities in diabetic hearts (*P* < 0.05; Additional file 1: Fig. S2a).

To further verify the effect of Adipsin on mitochondria, we performed in vitro experiments in which cardiomyocytes were treated with Ad-Adipsin and Ad-Control for 8 h and then assessed for 24 h mitochondrial stress in the presence or absence of PA (300 μmol/L). The basal respiration, maximal respiration, ATP production, and spare respiration capacities of PA-treated cardiomyocytes were significantly decreased, while the effects were alleviated by Ad-Adipsin (*P* < 0.05; Fig. 2d). Meanwhile, the mitochondrial membrane potential of Ad-Adipsin treated cells was significantly increased (*P* < 0.05; Additional file 1: Fig. S2b). This finding suggests a protective effect of Adipsin on myocardial contractile and mitochondrial function in DCM.

### Adipsin and Irak2 bind directly in cardiomyocytes

**Fig. 3:**
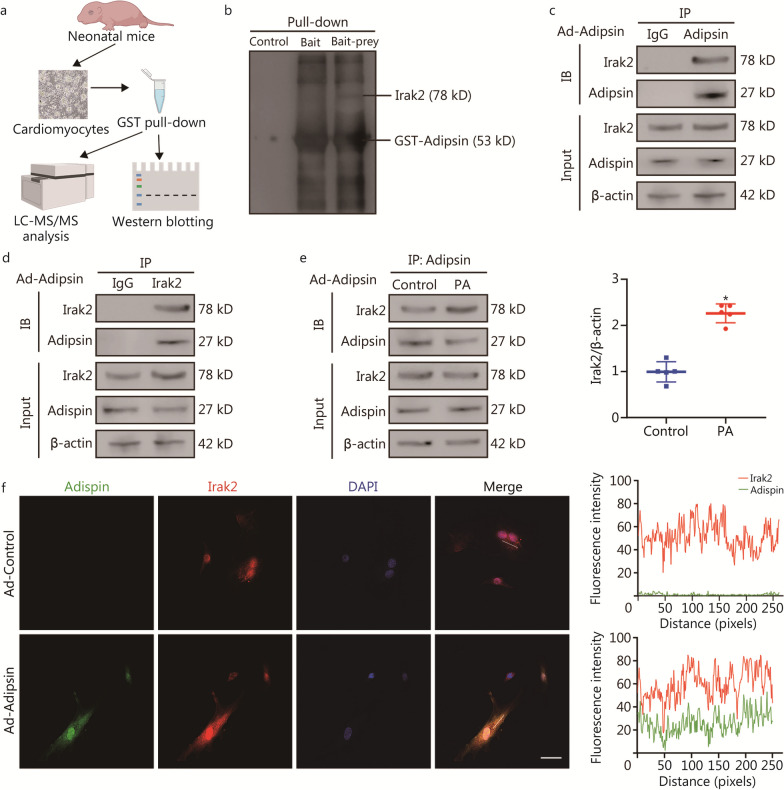
Adipsin and Irak2 bind directly in cardiomyocytes. **a** Schematic representation of the experimental protocol. The cardiomyocytes lysis prey protein was obtained by immobilizing GST-Adipsin with equilibrated glutathione agarose. Then, the eluted protein complexes were employed for mass spectrometry analysis and subsequent immunoblotting. **b** Proteins bound and pulled down by purified GST-Adipsin in cardiomyocytes. **c**, **d** Co-IP results indicating the interaction between Adipsin and Irak2 following the infection with Ad-Adipsin. **e** Co-IP results indicating the interaction between Adipsin and Irak2 with or without PA challenge following the infection with Ad-Adipsin (*n* = 5). **f** Colocalization images and analysis of Adipsin (green) and Irak2 (red) proteins in cardiomyocytes. Scale bar = 50 μm. Statistical significance was determined using a two-tailed Student’s *t* test. All data are represented with mean ± SD. ^*^P < 0.05 vs. Control. LC–MS/MS liquid chromatography-tandem mass spectrometry, Co-IP Co-immunoprecipitation, DAPI 4′,6-diamidino-2-phenylindole, Irak2 interleukin-1 receptor-associated kinase-like 2, PA palmitate, GST glutathione-S-transferase, IB immunoblotting

To investigate the mechanism by which Adipsin induces myocardial protection, we used the GST fusion protein sink technology (GST pull-down) to search for potential proteins that interact with Adipsin (Fig. 3a). We constructed a GST-Adipsin fusion protein to capture the protein(s) interacting with Adipsin in cardiomyocytes. Thus, the protein that interacts with adipsin, Irak2, was identified by liquid chromatography-tandem mass spectrometry (LC–MS/MS) analysis of captured prey proteins (Additional file 1: Table S6). We performed immunoblotting of prey proteins and found that Irak2 bands directly to Adipsin (Fig. 3b). In addition, Co-IP assays revealed a direct interaction between Adipsin and Irak2 in Ad-Adipsin treated cardiomyocytes (Fig. 3c, d, Additional file 1: Fig. S3). The interaction between Adipsin and Irak2 was enhanced in PA stimulated cardiomyocytes (2.26 ± 0.20 vs. 1.00 ± 0.22, *P* < 0.05; Fig. 3e). Moreover, the co-localization of Adipsin and Irak2 in cardiomyocytes was further verified by immunofluorescence (Fig. 3f). These data support that Adipsin enters cardiomyocytes and binds directly to Irak2.

### Overexpression of Adipsin inhibits mitochondrial translocation of Irak2

**Fig. 4:**
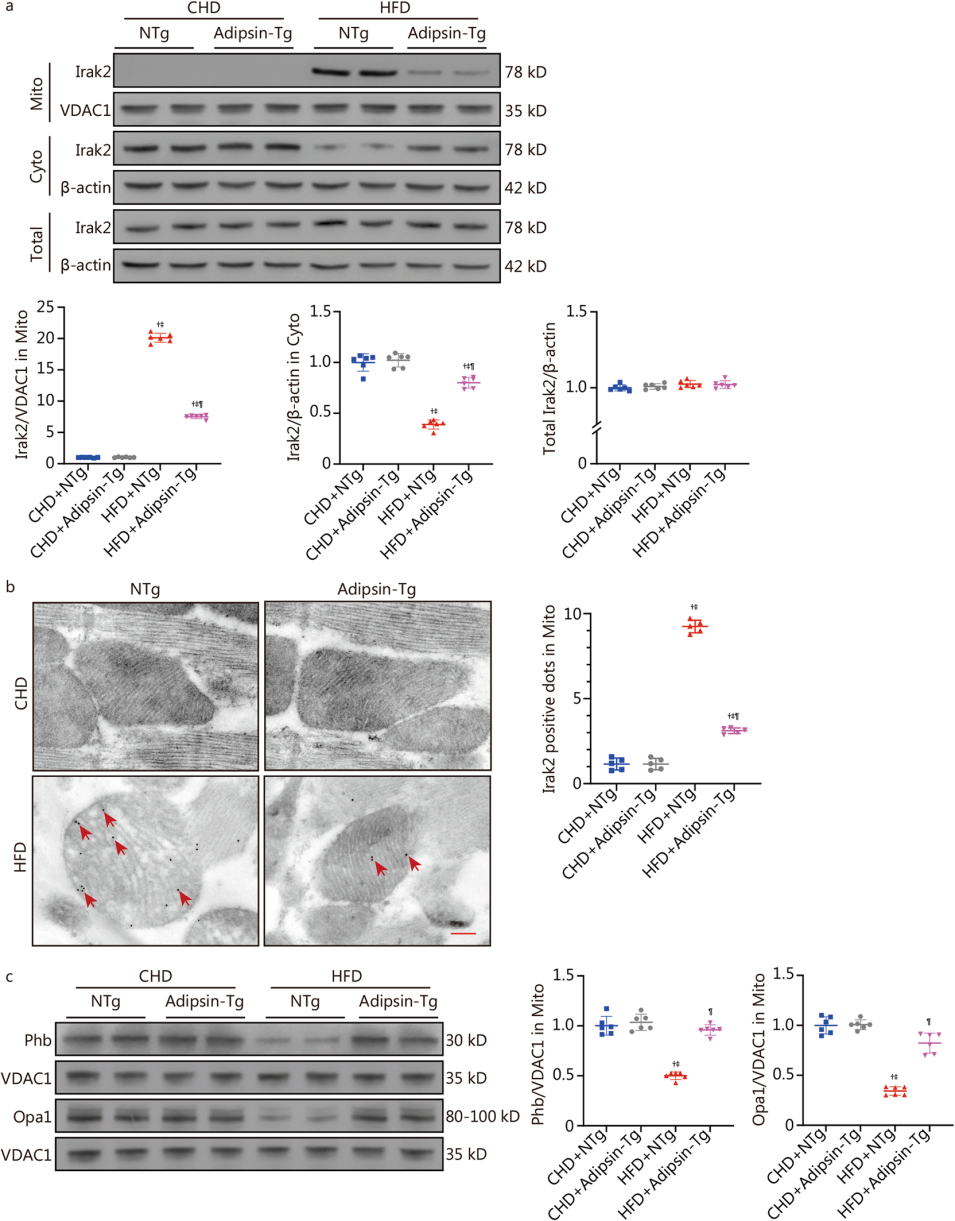
Overexpression of Adipsin inhibits mitochondrial translocation of Irak2. **a** Western blotting results and quantitative analysis of Irak2 expression in cytoplasm and mitochondria of cardiomyocytes in the CHD + NTg, CHD + Adipsin-Tg, NTg + HFD, and Adipsin-Tg + HFD groups (*n* = 6). **b** Electron microscopy images of Irak2 stained with immunogold and quantitative analysis of Irak2-positive dots (red arrow) in the myocardial mitochondria of NTg or Adipsin-Tg mice fed with CHD or HFD (*n* = 5). Scale bar = 200 nm. **c** Western blotting results and quantitative analysis of Phb and Opa1 in myocardial mitochondria of NTg or Adipsin-Tg mice fed with CHD or HFD (*n* = 6). Statistical significance was determined using one-way ANOVA. All data are represented with mean ± SD. ^†^*P* < 0.05 vs. CHD + NTg; ^‡^*P* < 0.05 vs. CHD + Adipsin-Tg; ^¶^*P* < 0.05 vs. HFD + NTg. Mito mitochondria, Cyto cytosol, CHD chow diet, HFD high-fat diet, Adipsin-Tg Adipsin tissue-specific transgenic mice, NTg nontransgenic mice, Irak2 interleukin-1 receptor-associated kinase-like 2, VDAC1 voltage-dependent anion channel 1, Phb prohibitin, Opa1 optic atrophy protein 1

To further evaluate the interaction between Adipsin and Irak2, we also examined changes in Irak2 expression in the myocardium of Adipsin-Tg mice. Our results showed that Adipsin overexpression decreased mitochondrial Irak2 expression and increased cytoplasmic Irak2 expression in DCM mouse myocardium, but the total Irak2 level was not changed (*P* < 0.05; Fig. 4a), indicating that Adipsin inhibited Irak2 translocation from cytoplasm to mitochondria. Similarly, overexpression of Adipsin reduced the number of Irak2-positive spots on mitochondria in diabetic hearts using immunocolloidal gold electron microscopy (*P* < 0.05; Fig. 4b). These observations further confirm that Adipsin reduces the mitochondrial translocation of Irak2. In addition, to explore the effect of Adipsin on Irak2, we examined Irak2 downstream molecules localized to myocardial mitochondria. Consistent with previous report [35], this study also demonstrated that Irak2 is exclusively expressed in the cytoplasm in the non-diabetes state, while Irak2 is able to translocate to the inner mitochondrial membrane and interact with prohibitin (Phb) and optic atrophy protein 1 (Opa1) upon PA challenge (Additional file 1: Fig. S4). Furthermore, the expression of Phb and Opa1 were downregulated in the myocardium of DCM mice induced by HFD. Adipsin overexpression significantly increased the expression of Phb and Opa1 in HFD-induced DCM mice (*P* < 0.05; Fig. 4c). The results further suggest that Phb and Opa1, as downstream of Irak2, are regulated by Adipsin overexpression in the animal model of DCM. In conclusion, Adipsin inhibits the mitochondrial translocation of Irak2 in DCM, thereby reducing the interaction between Irak2 and Phb-Opa1 on mitochondria and improving the structural integrity and function of mitochondria.

### Adipsin improves cardiac function, reduces mitochondrial cristae damage, and improves mitochondrial function through Irak2 signaling

**Fig. 5:**
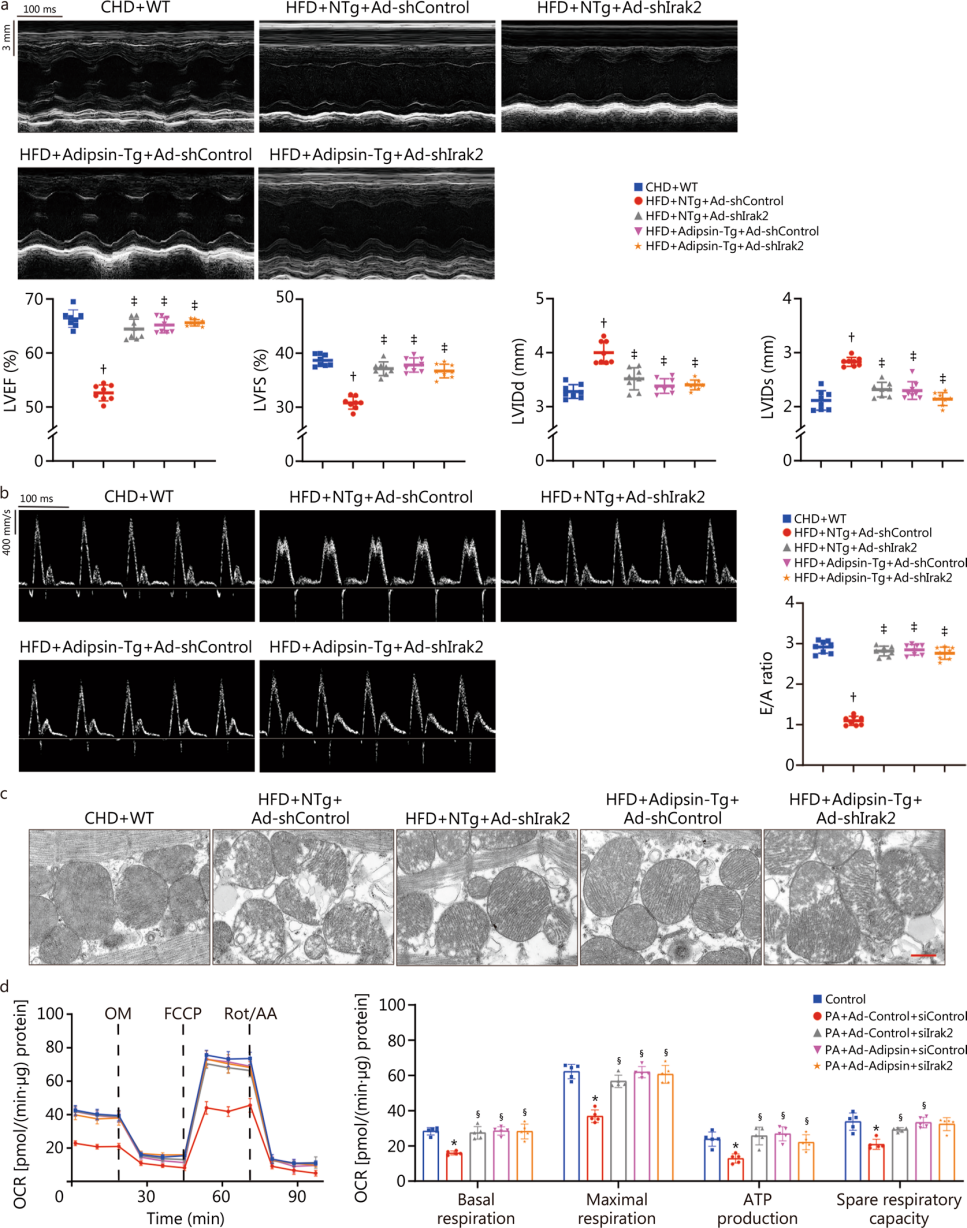
Adipsin improves cardiac function, reduces mitochondrial cristae damage, and improves mitochondrial function through Irak2 signaling pathway. **a** Echocardiographic results and quantitative analysis of echocardiographic data including LVEF, LVFS, LVIDd and LVIDs (*n* = 8). **b** Pulse-wave Doppler results and quantitative analysis of the E/A ratio (*n* = 8). **c** Transmission electron microscopic images of myocardium from different groups (*n* = 8). Scale bar = 500 nm. **d** OCR in cardiomyocytes from various treatment groups, and quantitative statistical analysis of OCR including basal respiration, ATP production, maximal respiration and spare respiration capacity. Statistical significance was determined using one-way ANOVA. All data are represented with mean ± SD. ^†^*P* < 0.05 vs. CHD + WT; ^‡^*P* < 0.05 vs. HFD + NTg + Ad-shControl; ^*^P < 0.05 vs. Con; ^§^*P* < 0.05 vs. PA + Ad-Control + siControl. CHD chow diet, HFD high-fat diet, Adipsin-Tg Adipsin tissue-specific transgenic mice, NTg nontransgenic mice, LVEF left ventricular ejection fraction, LVFS left ventricular fractional shortening, LVIDd end-diastolic left ventricular internal diameters, LVIDs end-systolic left ventricular internal diameters, E/A the ratio of mitral peak velocity of early filling (E) to mitral peak velocity of late filling (A), OCR oxygen consumption rate, OM oligomycin, FCCP fluoro-carbonyl cyanide phenylhydrazone, Rot/AA rotenone and antimycin A, PA palmitate, Irak2 interleukin-1 receptor-associated kinase-like 2, siControl control small interfering RNA

To further test whether the cardioprotective effect of Adipsin was mediated through Irak2, *Irak2* was knocked down in myocardial tissues. We found that mice receiving myocardial spot injection of Ad-shIrak2 successfully reduced Irak2 expression in myocardial tissues at the both mRNA and protein levels (*P* < 0.05; Additional file 1: Fig. S5a-c). Subsequently, mice were immediately placed on a HFD for 6 months and then examined for DCM-related phenotypes. Echocardiography analysis showed that compared with DCM mice, Adipsin-Tg DCM mice had significantly improved cardiac function, as indicated by increased LVEF, LVFS, and E/A ratio, as well as decreased LVIDd and LVIDs (*P* < 0.05; Fig. 5a, b). Interestingly, Adipsin overexpression did not further alleviate cardiac dysfunction in DCM mice under *Irak2* knockdown conditions, suggesting a downstream role of Irak2 in Adipsin-induced responses (*P* < 0.05; Fig. 5a, b). Our data further revealed the protective role of Adipsin overexpression against DCM-induced mitochondrial destruction. Similarly, overexpression of Adipsin attenuated mitochondrial destruction, enhanced mitochondrial complex I/II/III/IV/V activity in diabetic myocardial tissues. Consistently, mitochondrial damage was not further attenuated by Adipsin overexpression (*P* < 0.05; Fig. 5c, Additional file 1: Fig. S6a), and improved mitochondrial complex I/II/III/IV/V activities (*P* < 0.05; Additional file 1: Fig. S6b) after *Irak2* knockdown. Next, intracellular Irak2 was knocked down with siRNA prior to Ad-Adipsin and PA treatment of cardiomyocytes. Assessment of mitochondrial stress revealed a significant increase in basal respiration, maximal respiration, ATP production, and spare respiration capacity in cells treated with Ad-Adipsin in response to lipotoxic PA stimulation. Consistently, overexpression of Adipsin did not further improve oxidative phosphorylation (*P* < 0.05; Fig. 5d). Additionally, Adipsin overexpression did not further induce mitochondrial membrane potential benefit after *Irak2* knockdown (*P* < 0.05; Additional file 1: Fig. S6c). Taken together, these results indicate that Irak2 mitochondrial translocation plays an important role in DCM-induced contractile dysfunction, mitochondrial cristae damage, and mitochondrial injury in Adipsin-mediated myocardial protection.

### Adipsin improves cardiomyocyte FAO through the Irak2 signaling pathway

**Fig. 6:**
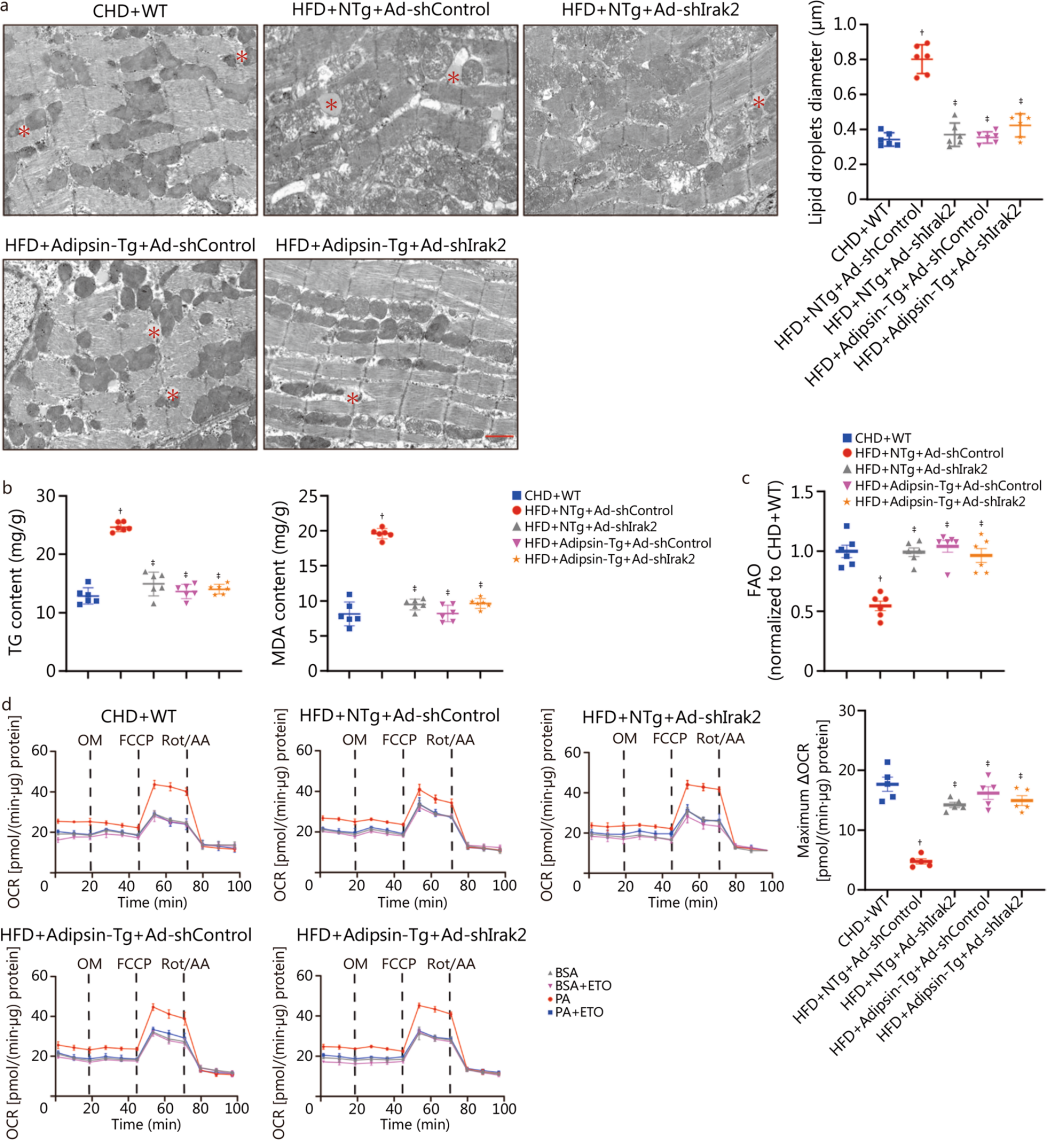
Adipsin improves cardiomyocyte FAO through the Irak2 signaling pathway. **a** Transmission electron microscopic images and quantitative analyses of mouse hearts depicting larger lipid droplets accumulation in various treatment groups (*n* = 6). Lipid droplets were marked by asterisks. Scale bar = 1 μm. **b** TG and MDA content in myocardial tissues (*n* = 8). **c** FAO evaluating with [3H]-oleic acid uptake (*n* = 6). **d** Oxidation pressure test of palmitate in cardiomyocytes from various treatment groups, and evaluation of FAO using quantitative analysis of maximum ΔOCR. Statistical significance was determined using one-way ANOVA. All data are represented with mean ± SD. ^†^*P* < 0.05 vs. CHD + WT; ^‡^*P* < 0.05 vs. HFD + NTg + Ad-shControl. CHD chow diet, HFD high-fat diet, Adipsin-Tg Adipsin tissue-specific transgenic mice, NTg nontransgenic mice, TG triglyceride, MDA malonaldehyde, FAO fatty acid β-oxidation, BSA bovine serum albumin, ETO etomoxir, PA palmitate, OCR oxygen consumption rate, OM oligomycin, FCCP fluoro-carbonyl cyanide phenylhydrazone, Rot/AA rotenone and antimycin A, △OCR oxygen consumption rate_[PA-(PA+ETO)]_—oxygen consumption rate_[BSA-(BSA+ETO)]_, Irak2 interleukin-1 receptor-associated kinase-like 2

Electron microscopic analysis showed that Adipsin overexpression significantly reduced the size of lipid droplets in the cardiac myocardium of DCM mice. After *Irak2* knockdown, overexpression of Adipsin did not further reduce myocardial lipid deposition (*P* < 0.05; Fig. 6a). The contents of TG and MDA were measured in myocardial tissues. The results showed that TG and MDA contents in myocardial tissue of DCM mice were significantly higher than those of WT mice, and these effects were reversed by Adipsin overexpression (*P* < 0.05). Interestingly, in the presence of *Irak2* knockdown, Adipsin overexpression did not further reduce TG and MDA contents in diabetic hearts (*P* < 0.05; Fig. 6b). These findings suggest that Adipsin inhibits the accumulation of lipids and their metabolites in the myocardium via Irak2.

Cardiomyocytes were isolated from diabetic mice overexpressing Adipsin (HFD + Adipsin-Tg) and corresponding controls. Our results indicated that FAO is significantly reduced in cardiomyocytes stressed by diabetes. Adipsin overexpression increased the level of FAO in cardiomyocytes. After *Irak2* knockdown, overexpression of Adipsin did not further increase the level of FAO in cardiomyocytes under diabetic stress (*P* < 0.05; Fig. 6c). To further test our hypothesis, we used the palmitic acid oxidative stress assay to assess FAO in diabetes-damaged cardiomyocytes. The dependence of cardiomyocytes on FAO was evaluated by measuring acute response to an FAO inhibitor (Etomoxir), also known as maximum ΔOCR. Our results indicated the maximum ΔOCR of cardiomyocytes was significantly reduced in HFD-fed mice, indicating that the extent of FAO was reduced in mice suffering from diabetic damage. In diabetic cardiomyocytes overexpressing Adipsin, the maximum ΔOCR of cells was significantly increased, indicating that Adipsin significantly improved FAO in diabetic hearts. As expected, Adipsin overexpression did not further increase maximum ΔOCR in diabetic hearts after *Irak2* knockdown (*P* < 0.05; Fig. 6d). These findings indicated that Adipsin reduced the accumulation of TG and MDA in diabetic myocardium, while improving FAO in diabetic cardiomyocytes. Irak2 may be involved in the development of DCM as a downstream regulator of Adipsin.

## Discussion

DCM is a major public health threat with few effective prevention and treatment options [36], so there is a need to elucidate the underlying mechanisms of this devastating comorbidity. Preservation of mitochondrial integrity and function has been shown to protect against the development of DCM [37, 38]. Here, we found that adipose tissue-specific overexpression of Adipsin improves cardiac dysfunction, mitochondrial structure disruption and mitochondrial damage in DCM. Adipsin, the first adipokine identified, is a serine protease homolog that is synthesized and secreted by adipocytes, and its synthesis is closely associated with metabolic status, especially the lipid profile [39, 40]. Previous studies have shown that Adipsin exerts a protective effect on pancreatic β cells, thereby improving hyperglycemia. At the same time, Adipsin levels were significantly reduced in obese mice [17, 41]. In parallel, our results showed that Adipsin levels were significantly reduced in DCM mice. Our further study showed that Adipsin had a cardioprotective effect to significantly improve cardiac function in DCM mice. These findings provide strong evidence for Adipsin as a novel target for DCM treatment.

DCM exposes the heart to metabolic stress, such as a high-fat environment, in our current experimental setting [42]. In order to adapt to the high-fat environment, the heart starts to switch to fatty acids as its main energy source [43]. With the continuous progress of metabolic pathology, excessive utilization and uptake of fatty acids/lipids cannot be compensated, leading to an imbalance of fatty acid supply and uptake, lipid overload (steatosis), accumulation of fatty acids and lipids in cardiomyocytes, causing lipotoxicity, poor FAO and impaired myocardial function [7, 9], which is consistent with our current experimental results in DCM mice. Interestingly, our data revealed that increased Adipsin levels significantly reduced myocardial TG accumulation and intracellular MDA content in cardiomyocytes of DCM mice, suggesting a beneficial role of Adipsin in intracellular lipid accumulation and lipotoxicity. Our study revealed that adipose tissue-specific overexpression of Adipsin promoted intracellular Adipsin content and improved FAO, suggesting the improvement of myocardial FAO may be involved in Adipsin induced responses in DCM. Not surprisingly, many preclinical studies have described the efficacy of targeting cardiac FAO to treat DCM [44, 45], suggesting the promise of FAO and metabolic adaptation in the treatment of DCM [42].

**Fig. 7:**
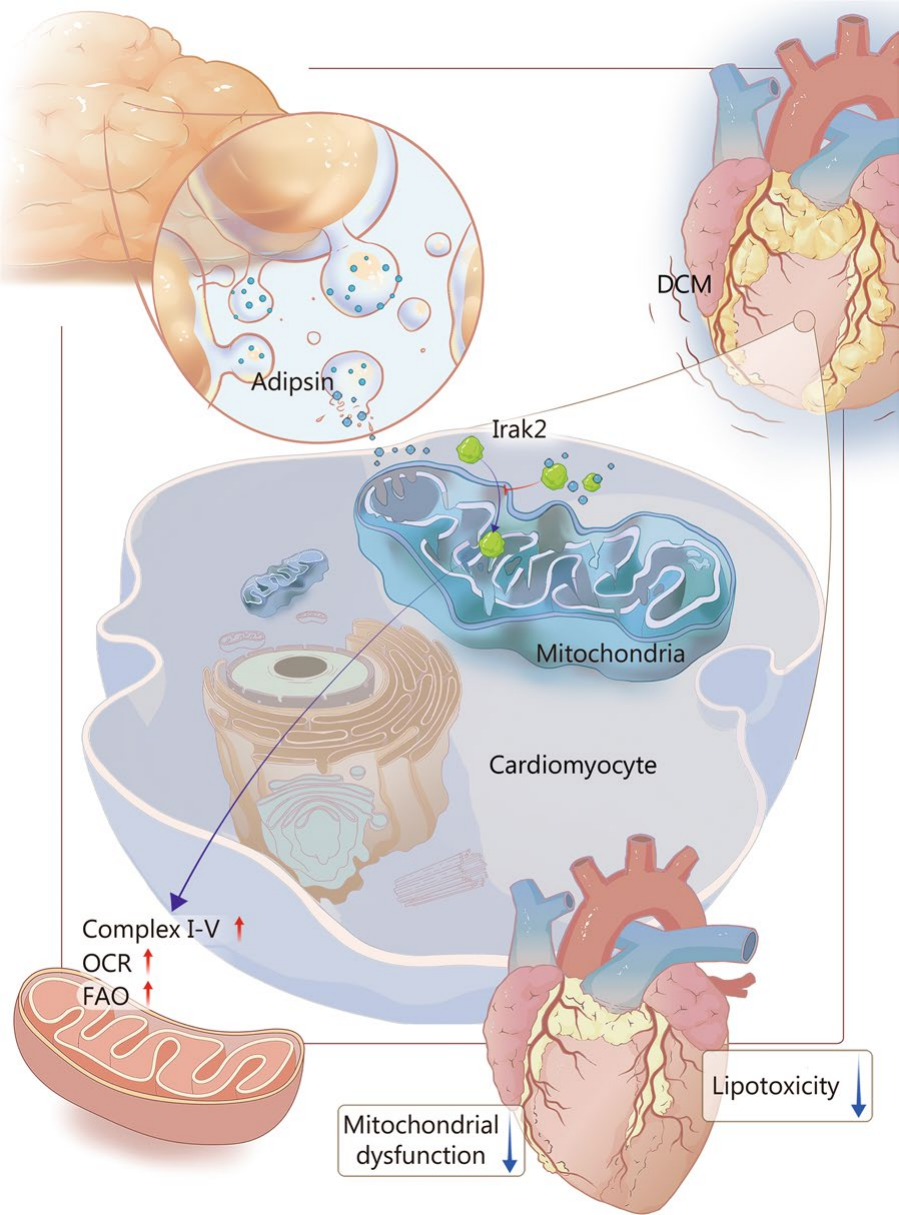
Schematic illustration of Adipsin-induced alleviation of diabetic cardiomyopathy (DCM) by inhibiting Irak2 mitochondrial translocation. Adipsin is secreted by adipose tissues and enters cardiomyocytes. Under HFD conditions, Adipsin enters cardiomyocytes to bind with Irak2 and inhibit its mitochondrial translocation. Reduction in Irak2 entry into mitochondria improves FAO, protects mitochondrial structure and function, reduces myocardial lipid accumulation, further improves cardiac function, and prevents DCM. HFD high-fat diet, OCR oxygen consumption rate, FAO fatty acid β-oxidation, Irak2 interleukin-1 receptor-associated kinase-like 2

To investigate the specific molecular mechanisms by which Adipsin protects cardiomyocytes, we found that Adipsin binds directly to Irak2 in cardiomyocytes. Irak2 is a member of the IL-1R-related kinase (IRAK) family [46]. Recent studies have shown that Toll-like receptor/IL-1 receptor (TLR/IL-1R) is closely related to cell metabolism, especially lipid accumulation, cellular growth, and ER stress, in which Irak2 plays a crucial regulatory role [47–49]. Importantly, defects in Irak2 or its kinase, in addition to demonstrating that translocation of Irak2 into the mitochondrial membrane space and inner membrane plays a role, also reduced HFD-induced metabolic disorders [24, 50]. In our study, Adipsin improved myocardial fatty acid metabolism and protected myocardial function by inhibiting Irak2 mitochondrial translocation. Irak2 has been shown to translocate into mitochondrial space and to locate to the inner mitochondrial membrane, where it interacts with Phb, resulting in the recruitment of Opa1 by Phb from cristae junctions, thereby disrupting the supercomplex and mitochondrial integrity [24]. Opa1 is an important molecule that regulates mitochondrial fusion and cristae integrity to control kinesin-related proteins, which favors mitochondrial cristae attachment [51]. Our results showed that increased Adipsin inhibits Irak2 mitochondrial translocation in diabetic myocardium, thereby eliminating the interaction between Irak2 and Phb-Opa1, reducing mitochondrial cristae damage, improving mitochondrial function, and myocardial function. Our further experiment showed that Irak2 plays an important role in the myocardial protection provided by Adipsin in DCM. Inhibition of Irak2 mitochondrial translocation by Adipsin can improve the structure and function of myocardial mitochondria, inhibit the accumulation of TG and MDA in cardiomyocytes, and improve FAO and the function of myocardial contraction, thereby blocking the pathogenesis of DCM. Importantly, we found that inhibition of the mitochondrial translocation of myocardial Irak2 through Adipsin action reduced the interaction of Irak2 with Phb-Opa1, resulting in improved mitochondrial integrity and function, and reduced TG and MDA accumulation in cardiomyocytes, leading to the improved FAO and myocardial function (Fig. 7).

Several limitations of this study should be considered. First, the induction of DCM using a HFD alone may not adequately mimic the situation of type 2 diabetes mellitus in humans. We need to elucidate the role of Adipsin in different types and stages of diabetes by inducing type 2 DCM with a combination of HFD and streptozotocin (STZ). Second, the present study showed that Adipsin could alleviate HFD-induced mitochondrial dysfunction and cardiac dysfunction. Since multiple cell types may be involved in the development and progression of DCM, further studies are needed to determine which specific cell types contribute to DCM cardiac remodeling. Third, our study illustrated that Adipsin effectively improved cardiomyocyte FAO. However, DCM is known to constitute complex substrate utilization and immune metabolic processes [42, 52], it is necessary to gain insight into the role of Adipsin on different substrate utilization and immune metabolism is warranted. Fourth, although we confirmed that Irak2 is an indispensable relevant molecule in the protective response induced by Adipsin, Irak2 may not be the only essential molecule for this process. Future studies should aim to elucidate the complex underlying mechanisms. Despite these obvious limitations, the present study provides novel therapeutic targets for DCM and compelling evidence for the mitochondrial protective properties of Adipsin.

## Conclusions

Our findings reveal an important cardioprotective role for Adipsin in DCM, where Adipsin provides a powerful beneficial complex response to myocardial and mitochondrial function in DCM. Importantly, Adipsin induced cardiac contractile benefits was closely associated with improved mitochondrial FAO and reduced myocardial lipotoxicity and lipid storage, thereby delaying the progression of DCM. Future work is needed to extend the possible cardioprotective properties of Adipsin in other cardiovascular diseases as well as to develop small molecules targeting Adipsin.

### Supplementary Information

**Additional file 1: Table S1** siRNA sequences for *Irak2*knockdown. **Table S2** Primer sequences for real-time quantitative-PCR. **Table S3** Primary antibodies used for Western blotting, immunoprecipitation, and immunohistochemistry studies. **Table S4** Blood glucose and body weight of experimental animals (mean ± SD, *n* = 9). **Table S5** Echocardiographic metrics of experimental animals (mean ± SD, *n* = 9). **Table S6** Mass spectrum analysis of GST-Adipsin-interacting proteins. **Fig. S1** Evaluation of Adipsin expression in myocardium and validation of successful construction of Adipsin-Tg mice. **Fig. S2** Adipsin overexpression improves mitochondrial function in the hearts of DCM. **Fig. S3** Interaction between Adipsin and Irak2 following administration of Ad-Adipsin in cardiomyocytes. **Fig. S4** Co-IP detected the interaction between Irak2 and Phb-Opa1 in myocardial mitochondria. **Fig. S5** Construction of AAV9-shIrak2 and verification of transfection effectiveness. **Fig. S6** Impact of *Irak2* knockdown on Adipsin-Tg induced protective effects of mitochondrial morphology and function.

## Data Availability

The datasets analysed during the current study are available from the corresponding author on reasonable request.

## Abbreviations

DCM: Diabetic cardiomyopathy
HFD: High-fat diet
CHD: Chow diet
LC–MS/MS: Liquid chromatography-tandem mass spectrometry
GST: Glutathione-S-transferase
Co-IP: Co-immunoprecipitation
Irak2: Interleukin-1 receptor-associated kinase-like 2
PA: Palmitate
BSA: Bovine serum albumin
TBS: Tris-buffered saline
LVIDs: End-systolic left ventricular internal diameters
LVIDd: End-diastolic left ventricular internal diameters
LVEF: Left ventricular ejection fraction
LVFS: Left ventricular fractional shortening
E/A: The ratio of mitral peak velocity of early filling (E) to mitral peak velocity of late filling (A)
WAT: White adipose tissue
BAT: Brown adipose tissue
VDAC1: Voltage-dependent anion channel 1
TF: Transferrin
MDA: Malondialdehyde
TG: Triglyceride
FAO: Fatty acid β-oxidation analysis
OCR: Oxygen consumption rate
TLR/IL-1R: Toll-like receptor/interleukin-1 receptor
Phb: Prohibitin
Opa1: Optic atrophy protein 1
Mito: Mitochondria
Cyto: Cytosol
STZ: Streptozotocin
MI: Myocardial infarction
ELISA: Enzyme-linked immunosorbent assay
IOD: Integrated option density
Adipsin-Tg: Adipsin tissue-specific transgenic mice
NTg: Nontransgenic mice
OM: Oligomycin
FCCP: Fluoro-carbonyl cyanide phenylhydrazone
Rot/AA: Rotenone and antimycin A
DAPI: 4′,6-Diamidino-2-phenylindole
IB: Immunoblotting
ETO: Etomoxir
2D Echo: Conventional echocardiography
E/e’: The ratio between mitral inflow and mitral annular excursion velocity during early diastole
Strain: Strain echocardiography
CS: Citrate synthase
