# Supplementary material for: Adipsin inhibits Irak2 mitochondrial translocation and improves fatty acid β-oxidation to alleviate diabetic cardiomyopathy
Source: Mil Med Res. 2023 Dec 11;10:63. doi: 10.1186/s40779-023-00493-5 (PMC10712050; doi:10.1186/s40779-023-00493-5)
Supplement: Supplementary file 1 — Additional file 1: Table S1 siRNA sequences for Irak2knockdown. Table S2 Primer sequences for real-time quantitative-PCR. Table S3 Primary antibodies used for Western blotting, immunoprecipitation, and immunohistochemistry studies. Table S4 Blood glucose and body weight of experimental animals (mean ± SD, n = 9). Table S5 Echocardiographic metrics of experimental animals (mean ± SD, n = 9). Table S6 Mass spectrum analysis of GST-Adipsin-interacting proteins. Fig. S1 Evaluation of Adipsin expression in myocardium and validation of successful construction of Adipsin-Tg mice. Fig. S2 Adipsin overexpression improves mitochondrial function in the hearts of DCM. Fig. S3 Interaction between Adipsin and Irak2 following administration of Ad-Adipsin in cardiomyocytes. Fig. S4 Co-IP detected the interaction between Irak2 and Phb-Opa1 in myocardial mitochondria. Fig. S5 Construction of AAV9-shIrak2 and verification of transfection effectiveness. Fig. S6 Impact of Irak2 knockdown on Adipsin-Tg induced protective effects of mitochondrial morphology and function. [file 40779_2023_493_MOESM1_ESM.pdf]

Table S1 siRNA sequences for *Irak2* knockdown

Primer sequence	Monomeric unit	Melting temperature (℃)	Molecular weight (g/mol)	Primer sequence
siIrak2-01-ss	GCACCUUUGCCGAUAUCUATT	21	53.9	6567.00
siIrak2-01-as	UAGAUAUCGGCAAAGGUGCTT	21	53.9	6733.20
siIrak2-02-ss	GCAGUCCAGUUUACCUGAATT	21	53.9	6630.10
siIrak2-02-as	UUCAGGUAAACUGGACUGCTT	21	53.9	6670.10
siIrak2-03-ss	GGAAGAUCAAGUCCAUGGATT	21	53.9	6756.20
siIrak2-03-as	UCCAUGGACUUGAUCUUCCTT	21	53.9	6544.00

Table S2 Primer sequences for real-time quantitative-PCR

Gene ID	Gene name	Primer sequence	Product length (bp)	Melting temperature (℃)
11537	*Adipsin* (*Cfd*)	Forward: CCCCGAGGCCGGATTCT	165	60.85
		Reverse: AGAGTCGTCATCCGTCACTC		58.91
108960	*Irak2*	Forward: TCTCGCCATGGCTTGCTACAT	161	62.19
		Reverse: GGACCCTCTCCATGGACTTGA		60.74
11461	*β-actin*	Forward: CCACCATGTACCCAGGCATT	240	60.03
		Reverse: CAGCTCAGTAACAGTCCGCC		60.74

Table S3 Primary antibodies used for Western blotting, immunoprecipitation, and immunohistochemistry studies

Antibody	Working dilutions	Catalog No.	Supplier
Adipsin	WB: 1/1000	A8117	ABclonal, Wuhan, China
	IHC/IF: 1/200	Sc-376015	Santa,California, USA
Irak2	WB: 1/1000	A2753	ABclonal, Wuhan, China
	IHC/IP: 1/200	Bs-1427R	Bioss, Beijing, China
Opa1	WB: 1/1000<br>IP:1/100	A9833	ABclonal, Wuhan, China
Phb	WB: 1/1000<br>IP:1/100	A0056	ABclonal, Wuhan, China
β-actin	WB: 1/200,000	AC026	ABclonal, Wuhan, China
VDAC1	WB: 1/1000	MABN504	Merk, New Jersey, USA
GST-Tag	WB: 1/1000	AE006	ABclonal, Wuhan, China
TF	WB: 1/1000	17435-1-AP	Proteintech, Wuhan, China

Irak2 interleukin-1 receptor-associated kinase-like 2, *Opa1* optic atrophy protein 1, *Phb* prohibitin, *VDAC1* voltage-dependent anion channel 1, *GST-Tag* glutathione-S-transferase-tag, *TF* transferrin

Table S4 Blood glucose and body weight of experimental animals (mean ± SD, $n = 9$)

Months	Body weight (g)		Blood glucose (mmol/L)	
	CHD	HFD	CHD	HFD
0	19.8 ± 2.1	19.2 ± 1.6	6.0 ± 1.1	6.2 ± 1.4
1	21.4 ± 1.5	21.8 ± 1.1	6.4 ± 1.0	6.8 ± 1.1
2	23.5 ± 2.0	26.4 ± 1.2*	6.7 ± 1.3	6.3 ± 1.6
3	24.2 ± 1.6	35.7 ± 1.7*	6.8 ± 1.3	10.5 ± 2.1*
4	25.6 ± 1.3	44.2 ± 1.2*	7.1 ± 1.4	15.6 ± 1.3*
5	26.3 ± 1.9	50.3 ± 1.0*	6.3 ± 1.3	20.5 ± 1.8*
6	28.2 ± 1.2	57.7 ± 1.7*	7.0 ± 1.3	23.1 ± 2.1*

*$P < 0.05$ vs. CHD; *CHD* chow diet, *HFD* high-fat diet

Table S5 Echocardiographic metrics of experimental animals (mean ± SD, $n = 9$)

Item	0 month		6 month	
	**CHD**	**HFD**	**CHD**	**HFD**
HR (bpm)	418 ± 18	415 ± 10	404 ± 25	420 ± 19
2D Echo (E/e’)	24.3 ± 2.6	21.01 ± 3.3	23.1 ± 5.5	56.7 ± 8.7*
Strain (%)				
Circumferential strain	-24.5 ± 2.6	-23.2 ± 3.5	-25.2 ± 4.4	-16.9 ± 5.3*
Longitudinal strain	-20.6 ± 3.5	-21.2 ± 4.3	-19.2 ± 5.4	-12.6 ± 4.12*
Radial strain	34.2 ± 1.5	33.4 ± 2.3	32.4 ± 1.72	26.62 ± 1.8*

*$P < 0.05$ vs. CHD; *CHD* chow diet, *HFD* high-fat diet, *2D Echo* conventional echocardiography, *E/e’* ratio between mitral inflow and mitral annular excursion velocity during early diastole, *Strain* strain echocardiography

Table S6 Mass spectrum analysis of GST-Adipsin-interacting proteins

Protein name	Unique peptides	Coverage	Molecule weight (kD)	MS score
Irak2	11	89.3	69.047	295.09
Hbb-b1	9	71.4	15.84	282.32
Hba	7	90.8	15.085	264.98
Myl2	13	76.5	18.864	236.95
Myl3	13	81.9	22.421	174.23

Cardiomyocyte proteins interacting with glutathione-S-transferase (GST)-Adipsin, the top 5 with high MS scores. *Irak2* interleukin-1 receptor-associated kinase-like 2, *Hbb-b1* hemoglobin subunit beta-1, *Hba* hemoglobin subunit alpha, *Myl2* myosin regulatory light chain 2, *Myl3* myosin light chain 3, *MS score* mass spectrometry score

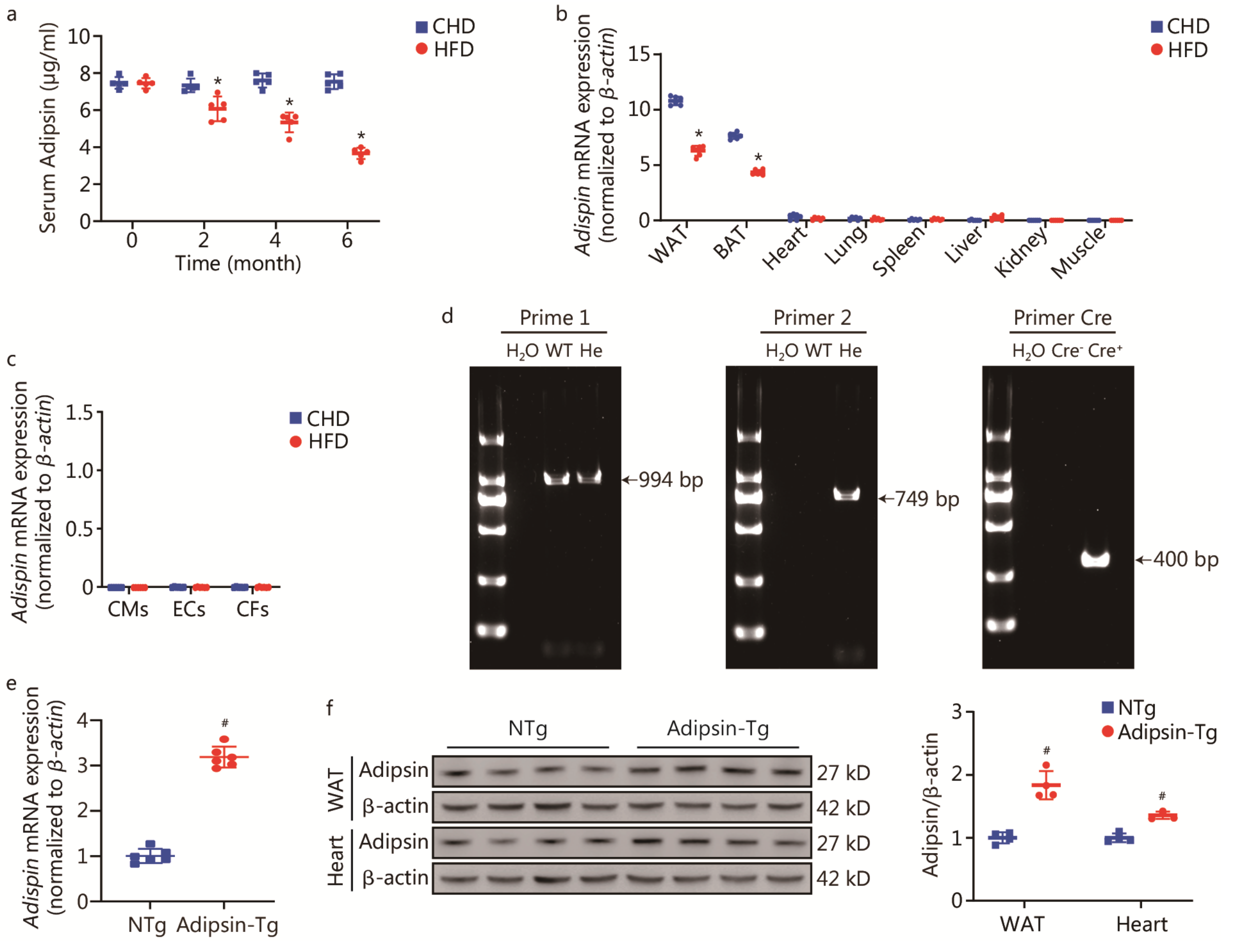

Fig. S1 Evaluation of Adipsin expression in myocardium and validation of successful construction of Adipsin-Tg mice. **a** ELISA quantitative analysis of serum Adipsin levels in the treatment groups at 0, 2, 4, and 6 months (n = 5). **b** Relative *Adipsin* mRNA expression in WAT, interscapular BAT, heart, lung, spleen, liver, kidney, and muscle tissues from mice with CHD- and HFD-fed for 6 months (n = 6). **c** Relative *Adipsin* mRNA expression in various cell types in the heart (n = 6). **d** Nucleic acid electrophoresis identifying the genotype of transgenic mice. **e** Relative *Adipsin* mRNA expression in Adipsin-Tg and NTg mice (n = 6). **f** Western blotting results and quantitative analysis of Adipsin expression in WAT and heart tissue lysates from Adipsin-Tg and NTg mice (n = 4). Statistical significance was determined using a two-tailed Student's t-test. All data are represented with mean ± SD. $^{*}P < 0.05$ vs. CHD; $^{\#}P < 0.05$ vs. NTg; Primer 1: 5'-TCAGATTCTTTTATAGGGGACACA-3' and 3'-TAAAGGCCACTCAATGCTCACTAA-5'; Primer 2: 5'-ACCTCCTCGCCCTTGCT -3' and 3'-GGCGTCTATACCCGAGTGTC-5'; Primer cre: 5'-ATTTGCCTGCATTACCGGTCG-3' and 3'-CAGCATTGCTGTCACTTGGTC-5'. CHD chow diet, HFD high-fat diet, WAT white adipose tissue, BAT brown adipose tissue, CMs cardiomyocytes, ECs endothelial cells, CFs cardiac

fibroblasts, WT wild type, He heterozygote, Adipsin-Tg Adipsin tissue-specific transgenic mice, NTg nontransgenic mice

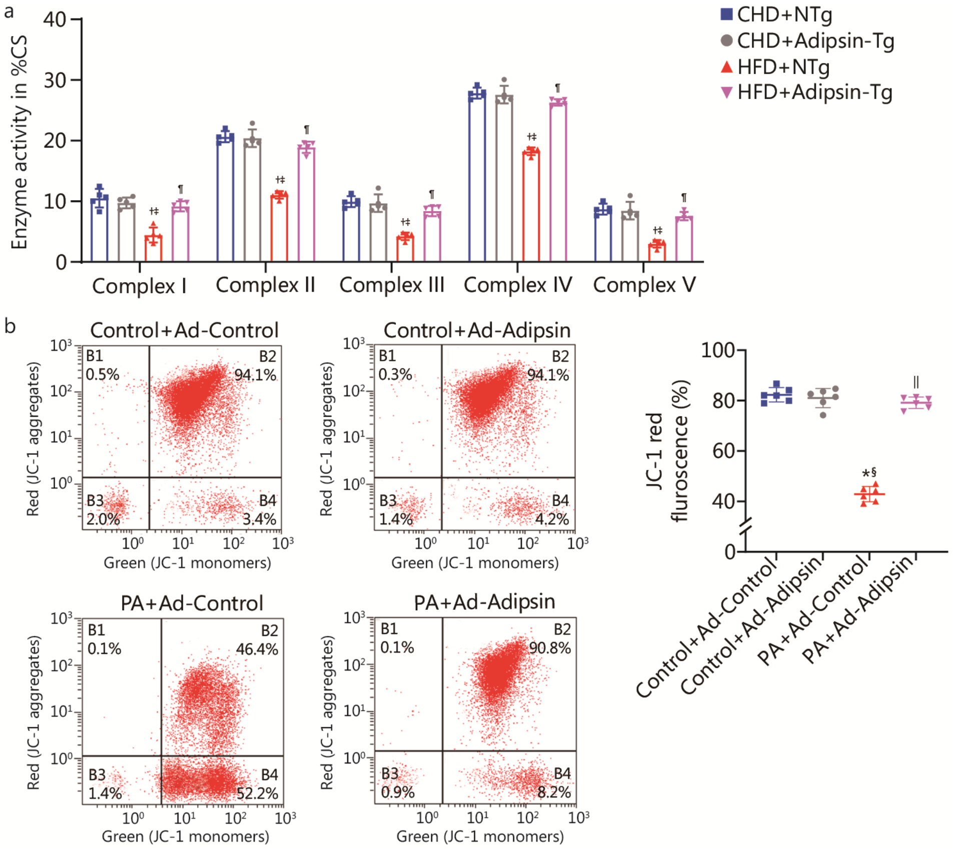

Fig. S2 Adipsin overexpression improves mitochondrial function in the hearts of DCM. **a** Complex I/II/III/IV/V activities of myocardium in various groups (*n* = 5). **b** Flow cytometry analysis and quantification of mitochondrial membrane potential using JC-1 staining in cardiomyocytes. Red fluorescence intensity was quantified (*n* = 6 wells). Statistical significance was determined using one-way ANOVA. Data are shown as mean ± SD. $^{\dagger}P < 0.05$ vs. CHD + NTg; $^{\ddagger}P < 0.05$ vs. CHD + Adipsin-Tg; $^{\P}P < 0.05$ vs. HFD + NTg; $^{*}P < 0.05$ vs. Control + Ad-Control; $^{\S}P < 0.05$ vs. Control + Ad-Adipsin; $^{\|}P < 0.05$ vs. PA + Ad-Control. PA palmitate, CHD chow diet, HFD high-fat diet, Adipsin-Tg Adipsin tissue-specific transgenic mice, NTg nontransgenic mice, CS citrate synthase

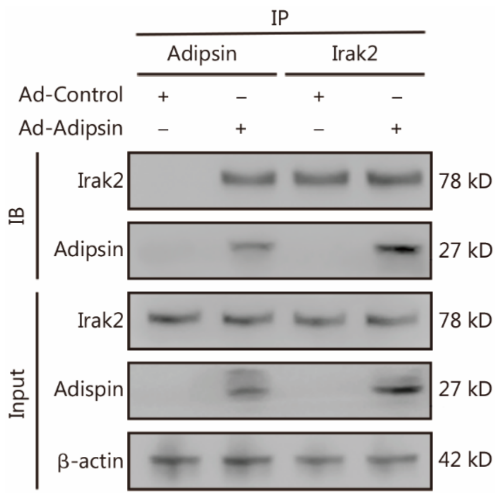

Fig. S3 Interaction between Adipsin and Irak2 following administration of Ad-Adipsin in cardiomyocytes. IP immunoprecipitation, Irak2 interleukin-1 receptor-associated kinase-like 2, IB immunoblotting

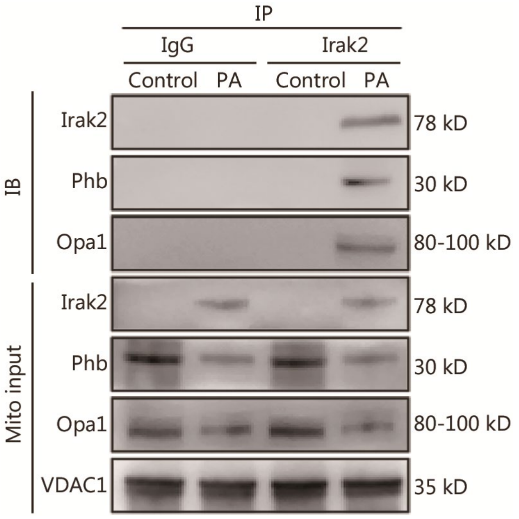

Fig. S4 Co-IP detected the interaction between Irak2 and Phb-Opa1 in myocardial mitochondria. IP immunoprecipitation, Irak2 interleukin-1 receptor-associated kinase-like 2, Phb prohibitin, Opa1 optic atrophy protein 1, VDAC1 voltage-dependent anion channel 1, Mito mitochondria, IB immunoblotting

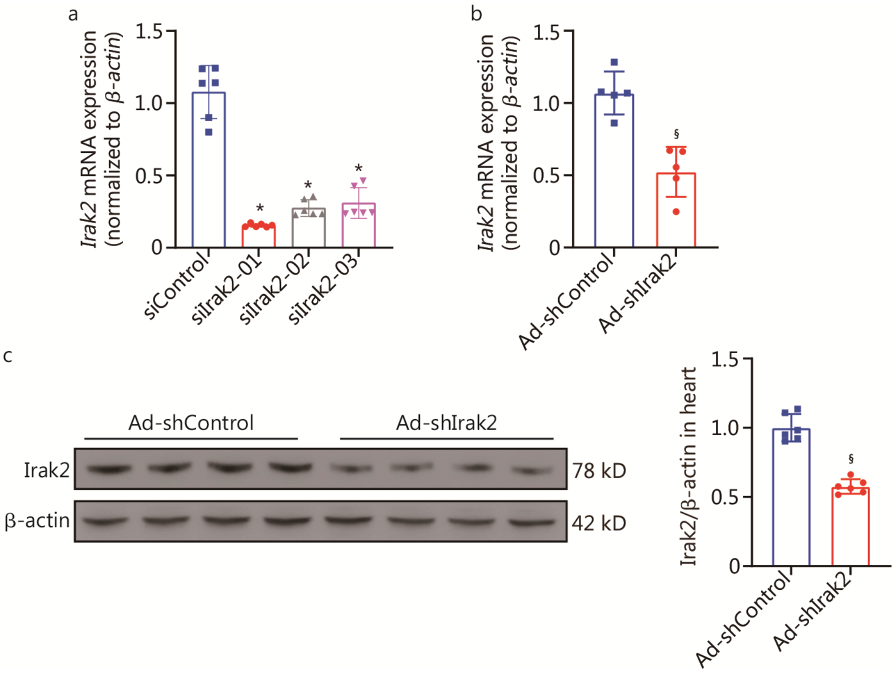

Fig. S5 Construction of AAV9-shIrak2 and verification of transfection effectiveness. **a** Relative *Irak2* mRNA expression following siIrak2 intervention in cardiomyocytes (n = 6). **b** Relative *Irak2* mRNA expression after intervention with Ad-shIrak2 in myocardium (n = 6). **c** Western blotting results and quantitative analysis of myocardial Irak2 expression after intervention with Ad-shIrak2 (n = 6). Statistical significance was determined by a two-tailed Student's t test. All data are represented with means ± SD. $^{*}P < 0.05$ vs. siControl; $^{\S}P < 0.05$ vs. Ad-shControl.

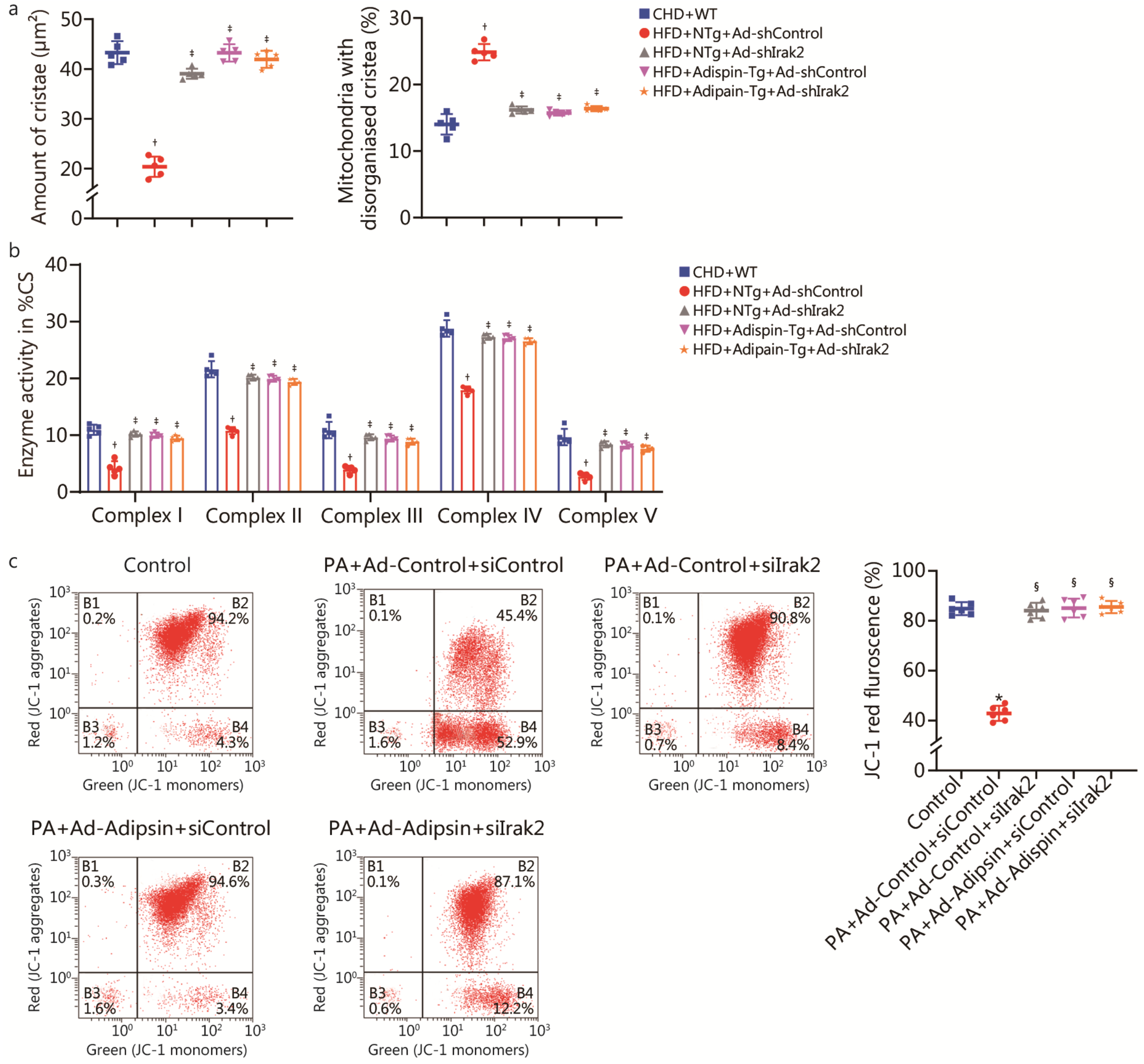

Fig. S6 Impact of *Irak2* knockdown on Adipsin-Tg induced protective effects of mitochondrial morphology and function. **a** Quantification of cristae amount per μm² and the proportion of mitochondria with disorganized cristae (n = 5). **b** Complex I/II/III/IV/V activities of myocardium in various groups (n = 5). **c** Flow cytometry analysis and quantification of mitochondrial membrane potential by JC-1 staining in cardiomyocytes. Red fluorescence intensity was quantified (n = 6 wells). Statistical significance was determined by one-way ANOVA. Data are shown as mean ± SD. $^{\dagger}P <$ 0.05 vs. CHD+WT; $^{\ddagger}P < 0.05$ vs. HFD + NTg + Ad-shControl; $^{*}P < 0.05$ vs. Control; $^{\S}P < 0.05$ vs. PA + Ad-Control + siControl. PA palmitate, CHD chow diet, HFD high-fat diet, Adipsin-Tg Adipsin tissue-specific transgenic mice, NTg nontransgenic mice, CS citrate synthase, Irak2 interleukin-1 receptor-associated kinase-like 2
